# Combined Renin Inhibition/(Pro)Renin Receptor Blockade in Diabetic Retinopathy- A Study in Transgenic (mREN2)27 Rats

**DOI:** 10.1371/journal.pone.0100954

**Published:** 2014-06-26

**Authors:** Wendy W. Batenburg, Amrisha Verma, Yunyang Wang, Ping Zhu, Mieke van den Heuvel, Richard van Veghel, A. H. Jan Danser, Qiuhong Li

**Affiliations:** 1 Division of Pharmacology, Vascular and Metabolic Diseases, Department of Internal Medicine, rasmus MC, GE Rotterdam, The Netherlands; 2 Department of Ophthalmology, College of Medicine, University of Florida, Gainesville, Florida, United States of America; Justus-Liebig-University Giessen, Germany

## Abstract

Dysfunction of renin-angiotensin system (RAS) contributes to the pathogenesis of diabetic retinopathy (DR). Prorenin, the precursor of renin is highly elevated in ocular fluid of diabetic patients with proliferative retinopathy. Prorenin may exert local effects in the eye by binding to the so-called (pro)renin receptor ((P)RR). Here we investigated the combined effects of the renin inhibitor aliskiren and the putative (P)RR blocker handle-region peptide (HRP) on diabetic retinopathy in streptozotocin (STZ)-induced diabetic transgenic (mRen2)27 rats (a model with high plasma prorenin levels) as well as prorenin stimulated cytokine expression in cultured Müller cells. Adult (mRen2)27 rats were randomly divided into the following groups: (1) non-diabetic; (2) diabetic treated with vehicle; (3) diabetic treated with aliskiren (10 mg/kg per day); and (4) diabetic treated with aliskiren+HRP (1 mg/kg per day). Age-matched non-diabetic wildtype Sprague-Dawley rats were used as control. Drugs were administered by osmotic minipumps for three weeks. Transgenic (mRen2)27 rat retinas showed increased apoptotic cell death of both inner retinal neurons and photoreceptors, increased loss of capillaries, as well as increased expression of inflammatory cytokines. These pathological changes were further exacerbated by diabetes. Aliskiren treatment of diabetic (mRen2)27 rats prevented retinal gliosis, and reduced retinal apoptotic cell death, acellular capillaries and the expression of inflammatory cytokines. HRP on top of aliskiren did not provide additional protection. In cultured Müller cells, prorenin significantly increased the expression levels of IL-1α and TNF-α, and this was completely blocked by aliskiren or HRP, their combination, (P)RR siRNA and the AT1R blocker losartan, suggesting that these effects entirely depended on Ang II generation by (P)RR-bound prorenin. In conclusion, the lack of effect of HRP on top of aliskiren, and the Ang II-dependency of the ocular effects of prorenin in vitro, argue against the combined application of (P)RR blockade and renin inhibition in diabetic retinopathy.

## Introduction

Diabetic retinopathy (DR) is the most common diabetic microvascular complication and the leading cause of severe vision loss in people under the age of sixty [1]. The prevalence of DR increases with the duration of diabetes, and nearly all individuals with type 1 diabetes and more than 60% of those with type 2 diabetes have some form of retinopathy after 20 years [2], [3].

Several molecular, biochemical and hemodynamic pathways have been identified to contribute to the pathogenesis of DR [4]. Hyperactivity of the renin-angiotensin system (RAS), resulting in elevated concentrations of its principal effector peptide, angiotensin (Ang) II, plays a key role in activating pathways leading to increased vascular inflammation, oxidative stress, endothelial dysfunction and tissue remodeling in variety of conditions, including diabetes and its associated complications [5], [6]. As a result, RAS inhibitors are first-line therapeutic agents for treating patients with cardiovascular diseases, cardiometabolic syndrome and diabetes. Although the RAS was classically considered a circulating system having general systemic effects, it is now recognized that there are also local tissue RASs, for instance in the retina [7]. Activation of such local RASs may contribute to end-organ damage in diabetes and associated complications [7], [8].

It has long been recognized that diabetes with microvascular complications is associated with increased plasma prorenin [9], [10]. Relative to albumin, ocular fluids contain higher concentrations of prorenin than plasma, and ocular prorenin increases even further in diabetic patients with proliferative retinopathy [10]–[12]. This has led to the suggestion that prorenin may be a marker of diabetic complications [10]. Elevated prorenin may contribute to diabetic complications by binding to its putative receptor, the so-called (pro)renin receptor ((P)RR). Such binding activates prorenin, leading to increased angiotensin generation in target tissue and subsequent signaling by the classic RAS pathway [13]. In addition, binding of (pro)renin to the (P)RR also directly initiates a cascade of signaling events involving mitogen-activated protein kinases such as ERK1/2 and p38, that are known to be associated with profibrotic and proliferative actions independent of Ang II [13], [14]. The role of (P)RR in end-organ damage and diabetic complications is supported by several studies demonstrating that an inhibitor of (P)RR, a peptide derived from the prosegment of prorenin, the so-called handle-region peptide (HRP), afforded renal and cardiovascular protection [15], [16], presumably by inhibiting the binding of prorenin to the (P)RR [17]. HRP has also been shown to be beneficial in ocular pathologies [18]–[21].

Aliskiren is the first renin inhibitor that blocks the activity of renin, a key rate-limiting enzyme in the first step of the RAS cascade [22]. It has shown high efficacy not only in controlling blood pressure [23], but also in renal and cardiovascular protection [24], [25]. It is also effective in patients with metabolic syndrome, obesity and diabetes [26], [27], as well as in experimental atherosclerosis [28], [29]. Although aliskiren is able to bind to (P)RR-bound renin and prorenin [30]–[32], its role in (P)RR-mediated Ang II-independent signaling remains controversial with conflicting reports [33], [34]. We have previously shown that HRP unexpectedly counteracted some of the beneficial effects of aliskiren [35], [36]. In the present study, we investigated the effects of aliskiren on diabetes-induced retinopathy in a transgenic rat model overexpressing the mouse renin gene, i.e., the (mRen2)27 rat (Ren2 rat). This rat exhibits significantly elevated prorenin and Ang II levels, and displays increased PRR expression and severe hypertension [37]–[40]. After streptozotocin injection, these animals develop a diabetic phenotype that closely mimics that in diabetic patients, characterized by high prorenin levels, retinal pathology, vascular dysfunction and nephropathy [41], [42]. We also examined the effects of HRP on top of renin inhibition with aliskiren. Our data show that renin inhibition with aliskiren reduced both neuronal and vascular pathologies in the diabetic Ren2 rat retina, and that HRP on top of aliskiren did not provide additional protection, nor counteracted the beneficial effects of aliskiren.

## Materials and Methods

### Animals and treatment groups

Adult heterozygous Ren2 rats (400–500 g) with a Sprague–Dawley (SD) background [36] were used in this study. Rats were housed in individual cages and maintained on a 12-h light/dark cycle, having access to standard laboratory rat chow and water ad libitum. All procedures were performed under the regulation and approval of the Animal Care Committee of the Erasmus MC. Blood pressure was measured by radiotelemetry transmitters that were implanted as described before [43] two weeks prior to diabetes induction. To induce diabetes, rats were fasted overnight and administered streptozotocin (STZ; 55 mg/kg i.p., Sigma–Aldrich, Zwijndrecht, The Netherlands). Blood glucose levels were checked by tail incision (Precision Xceed, Abbott, Zwolle, The Netherlands). Only rats with a glucose level >15 mmol/L were considered diabetic, and they subsequently received 2–4 U insulin per day (Levemir, Novo Nordisk, Denmark). Aliskiren (a gift of Novartis, 10 mg/kg per day) with (n = 8) or without (n = 7) rat HRP (NH_2_- RILLKKMPSV-COOH, 1 mg/kg per day, Biosynthan, Berlin, Germany), or vehicle (saline, n = 8) were administered for 3 weeks by osmotic minipumps (2ML4 ALZET, Cupertino, USA) implanted subcutaneously two weeks after diabetes induction. In the animals receiving two drugs, two separate minipumps were implanted at both sides of the body. Eyes were collected at the end of the treatment period. For comparison, eyes were also collected from 9 untreated nondiabetic Ren2 rats, and 5 age-matched untreated SD rats.

### Retinal histology and immunofluorescence

Eyes were fixed in 4% paraformaldehyde freshly made in phosphate-buffered saline (PBS) at 4°C overnight. Eyecups were cryoprotected in 30% sucrose/PBS for several hours or overnight prior to quick freezing in optical cutting temperature (OCT) compound. Then 12 µm thick sections were cut at –20 to –22°C. A rabbit polyclonal antibody against glial fibrillary acidic protein (GFAP, 1∶1000, Sigma, St. Louis, MO), was used. The secondary antibody (conjugated with Alexa594) was from Molecular Probes (Invitrogen, Carlsbad, CA) and used according to the manufacturer’s instruction. Sections were coverslipped using VECTASHIELD (Vector Laboratories, Burlingame, CA), and examined with a Zeiss (AxionVision, Carl Zeiss MicroImaging, Inc, NY) microscope equipped with epifluorescence illumination and a high resolution digital camera. For morphological analysis, eyes were fixed in 10% formalin solution, processed for paraffin embedding, sectioned at a thickness of 4 µm, and stained with hematoxylin and eosin (H&E). For quantitative measurement of retinal ganglion cell (RGC) density, the number of nuclei in the RGC layer was counted from at least 10 sections from each eye, 4 eyes from each group.

### In situ cell death detection

To detect apoptotic cells, an In Situ Cell Death Detection Kit based on TUNEL technology (In Situ Cell Death Detection Kit, TMR red, Roche Applied Science, Indianapolis, IN, USA) was used according to the instructions supplied by the manufacturer.

### Retinal vascular preparation and H&E staining

Retinal vasculature was prepared using method as described previously [44]. Briefly, eyes were fixed in 4% paraformaldehyde freshly made in PBS overnight. Retinas were dissected out from the eyecups and digested in 3% trypsin (GIBCO-BRL) for 2–3 hours at 37°C. Retinal vessels were separated from other retinal neuronal cells by gentle shaking and manipulation under a dissection microscope. The vessels were then mounted on a clean slide, allowed to dry, and stained with PAS-H&E (Periodic Acid Solution- Hematoxylin, Gill No.3, Sigma, St. Louis, MO) according to the instruction manual. After staining and washing in water, the tissue was dehydrated and mounted in permount mounting media. The prepared retinal vessels were photographed using a Zeiss microscope equipped with a high resolution digital camera (AxioCam MRC5, Zeiss Axionvert 200). Six to eight representative, non-overlapping fields from each quadrant of the retina were imaged. Acellular capillaries were counted from images for each retina, and expressed as number of acellular vessels per mm^2^.

### RT-PCR of inflammatory cytokines

Total RNA was isolated from freshly dissected retinas using Trizol Reagent (Invitrogen, Carlsbad, CA) according to the manufacturer’s instruction. Reverse transcription was performed using Enhanced Avian HS RT-PCR kit (Sigma, St. Louis, MO) following the manufacturer’s instructions. Real time PCR was carried out on a real time thermal cycler (iCycler, Bio-Rad Life Sciences) using iQTM Syber Green Supermix (Bio-Rad Life Sciences). The threshold cycle number (Ct) for real-time PCR was set by the cycler software. Optimal primer concentration for PCR was determined separately for each primer pair. The primer sequences used in this study are shown in Table 1. Each reaction was run in duplicate or in triplicate, and reaction tubes with target primers and those with GAPDH/actin primers were always included in the same PCR run. To test the primer efficiencies, the one-step RT-PCR was run with each target primer. Relative quantification was achieved by the comparative 2-ΔΔCt method1. The relative increase/decrease (fold-induction/repression) of mRNA of target X in the experimental group (EG) was calculated using the control group as the calibrator: 2-ΔΔCt, where ΔΔ Ct is {Ct.x [EG] -Ct. GAPDH [EG]} - {Ct.x [control] - Ct. GAPDH [control]}.

**Table 1:** Primers used for Real-Time RT-PCR analysis.

Gene name	Accession #	Sequences
Rat VEGF	NM_031836	Forward: 5′ -TGCACCCACGACAGAAGGGGA-3′
		Reverse: 5′-TCACCGCCTTGGCTTGTCACAT-3′
Rat MCP1	NM_031530	Forward: 5′ -GCAGCAGGTGTCCCAAAGAAGCT-3′
		Reverse: 5′-AGAAGTGCTTGAGGTGGTTGTGGAA-3′
Rat ICAM1	NM_012967	Forward: 5′ -CCCCACCTACATACATTCCTAC-3′
		Reverse: 5′-ACATTTTCTCCCAGGCATTC-3′
Rat TNFα	NM_012675	Forward: 5′ -CCTTATCTACTCCCAGGTTCTC-3′
		Reverse: 5′-TTTCTCCTGGTATGAAATGGC-3′
Rat GAPDH	AF106860	Forward: 5′ -TGCTGGGGCTGGCATTGCTC-3′
		Reverse: 5′- CCCCAGGCCCCTCCTGTTGT-3′
Human IL-1α	NM_000575	Forward: 5′-ATCAGTACCTCACGGCTGCT-3′
		Reverse: 5′-TGGGTATCTCAGGCATCTCC-3′
Human TNFα	NM_000594	Forward: 5′-ATCTACTCCCAGGTCCTCTTCAA-3′
		Reverse: 5′-GCAATGATCCCAAAGTAGACCT-3′
Human β actin	NM_001101	Forward: 5′-GCAGGAGTATGACGAGTCCG-3′
		Reverse: 5′-AGGGACTTCCTGTAACAATGC-3′

### Cell culture and treatment

Müller cells (a kind gift of Dr. G. Astrid Limb, UCL Institute of Ophthalmology, London, United Kingdom) [45] were cultured in 24-well plates with Dulbecco’s Modified Eagle Medium (DMEM) (Thermo Fisher Scientific, Waltham, MA) containing 10% fetal bovine serum (FBS) (Thermo Fisher Scientific, Waltham, MA). The ON-Target plus (P)RR siRNA and scrambled control siRNA were from (Thermo Fisher Scientific, Waltham, MA). Different concentrations and incubation times of (P)RR-siRNA were pre-tested to determine the optimal concentration and incubation time according to the manufacturer’s recommendation. Cells grown to 70% confluence in the serum medium were replaced by Opti-MEM I-reduced serum medium (Life Technologies, Carlsbad, CA), and the ON-Target plus (P)RR siRNA or control siRNA, or vehicle only was added into the medium at a final concentration of 30 nmol/L. After 24-hour incubation with the siRNA, the cells were replaced with serum-free DMEM medium (Thermo Fisher Scientific, Waltham, MA) for 2 hours. Following the serum starvation, the cells were either untreated (control) or treated with prorenin (100 nmol/L) alone, prorenin (100 nmol/L) + HRP (10 µmol/L, GenScript), prorenin + aliskiren (10 µmol/L), prorenin + HRP (10 µmol/L) + aliskiren (10 µmol/L), and prorenin (100 nmol/L) + losartan (10 µmol/L, according to previous studies [46]). After the 6 hour incubation, cells were harvested and total RNA was extracted, real time RT-PCR was performed and analyzed using the methods described previously.

### Statistics

All values are presented as mean ± SD. Paired Student’s t-test was used to assess significance between two groups. One-way ANOVA followed by the post hoc Tukey (Fisher’s protected least significant difference) test was used to assess statistical significance between multiple groups. Differences were considered significant at p<0.05.

## Results

### Induction of diabetes mellitus and blood pressure

Blood glucose levels and mean arterial pressure (MAP) have been reported before [36] . In short, Ren2 rats had a normal blood glucose level, identical to that in Sprague–Dawley (SD) rats. STZ-induced diabetes mellitus increased blood glucose levels ∼5-fold, and treatment with aliskiren or aliskiren+HRP did not alter this. Aliskiren lowered MAP in Ren2 rats from 123±4 mmHg to 104±5 mm Hg, and this effect was unaltered by simultaneous administration of HRP [47].

### Retinal gliosis

**Figure 1:**
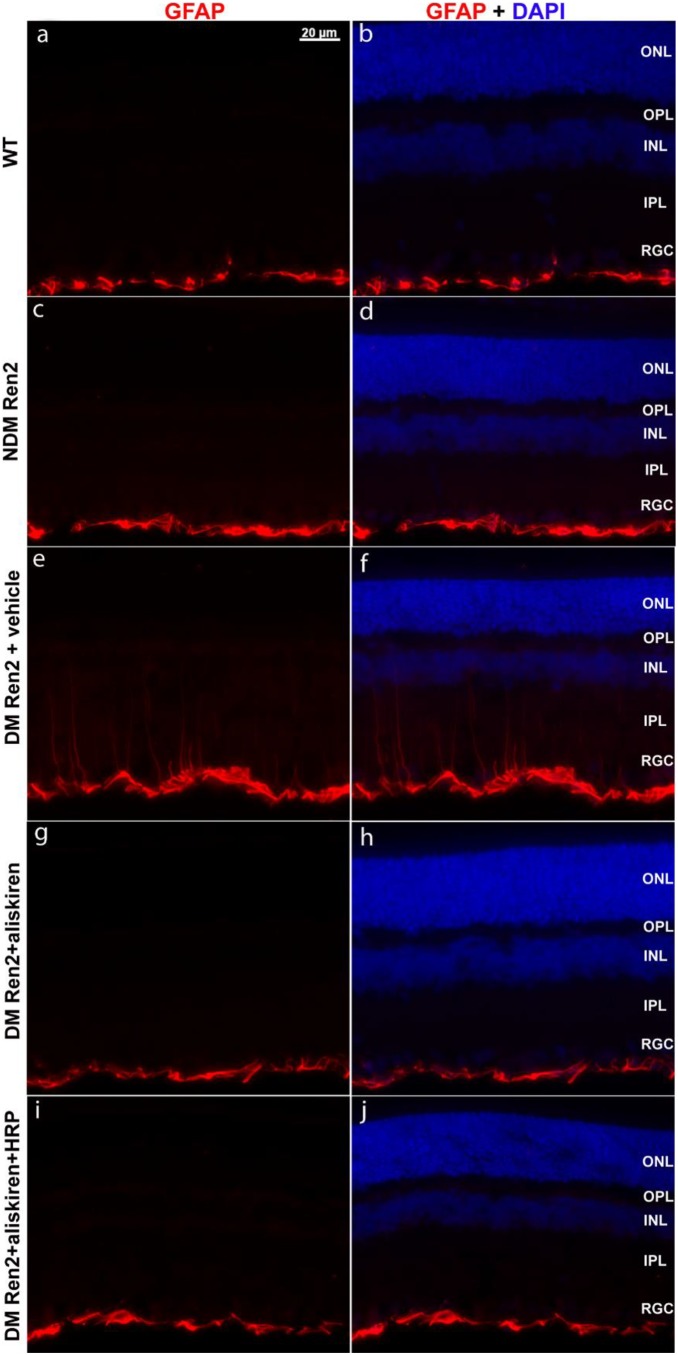
Immunofluorescence detection of GFAP expression in the retinas of wildtype (WT) Sprague–Dawley rats, non-diabetic (NDM) Ren2, diabetic (DM) Ren2 rats treated with vehicle, aliskiren or aliskiren+HRP. ONL: outer nuclear layer; OPL: outer plexiform layer; INL: inner nuclear layer; IPL: inner plexiform layer; RGC: retinal ganglion cell layer.

Retinal gliosis, a salient feature of the retina under stress or injury including diabetic retinopathy, is characterized by elevated expression of the intermediate filament, glial fibrillary acidic protein (GFAP) in Müller cells and astrocytes. GFAP is normally predominantly expressed in astrocytes in the inner retinal fiber layer (Figure 1a, b). A slight increase of GFAP expression was observed in astrocytes of the retina of non-diabetic Ren2 rats, but this was not different from the wildtype SD rat retina (Figure 1c, d). GFAP expression was elevated in the diabetic Ren2 rat retina (Figure 1e, f), and aliskiren prevented this elevation, both without (Figure 1g, h) and with HRP (Figure 1i, j).

### Apoptosis and RGC loss

**Figure 2:**
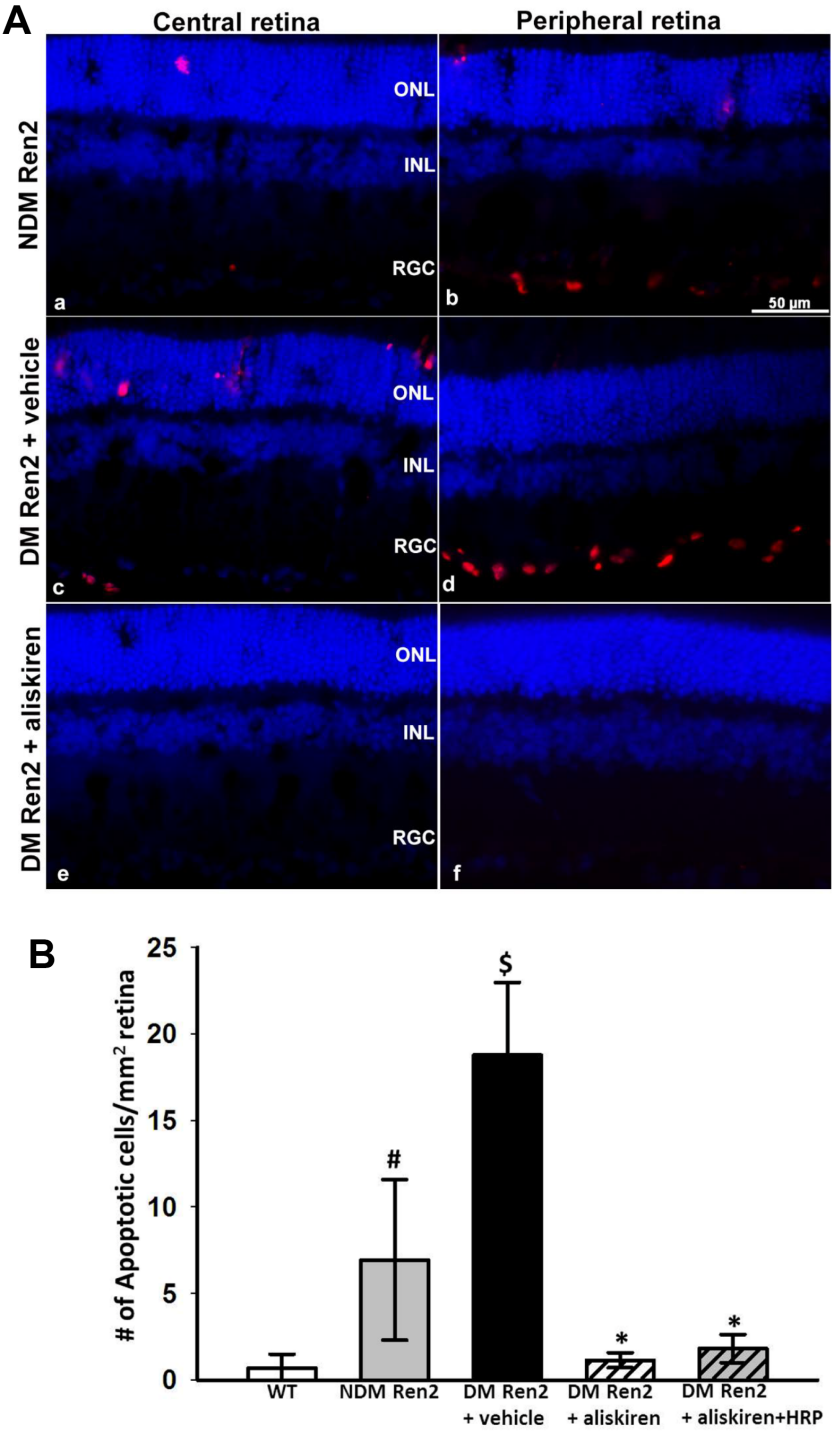
In situ cell death detection in the retina. A, Apoptotic cells were detected in-diabetic (NDM) Ren2 rats (a & b), diabetic (DM) Ren2 rats treated with vehicle (c & d), and aliskiren (e & f). ONL: outer nuclear layer; INL: inner nuclear layer; RGC: retinal ganglion cell layer. B, Quantitative measurement of apoptotic cells detected by TUNEL assay. #p<0.01 (vs. wildtype (WT) Sprague–Dawley rats); $p<0.01 (vs. NDM Ren2 and WT); *p<0.01 (vs. NDM Ren2 and DM Ren2+ vehicle).

**Figure 3:**
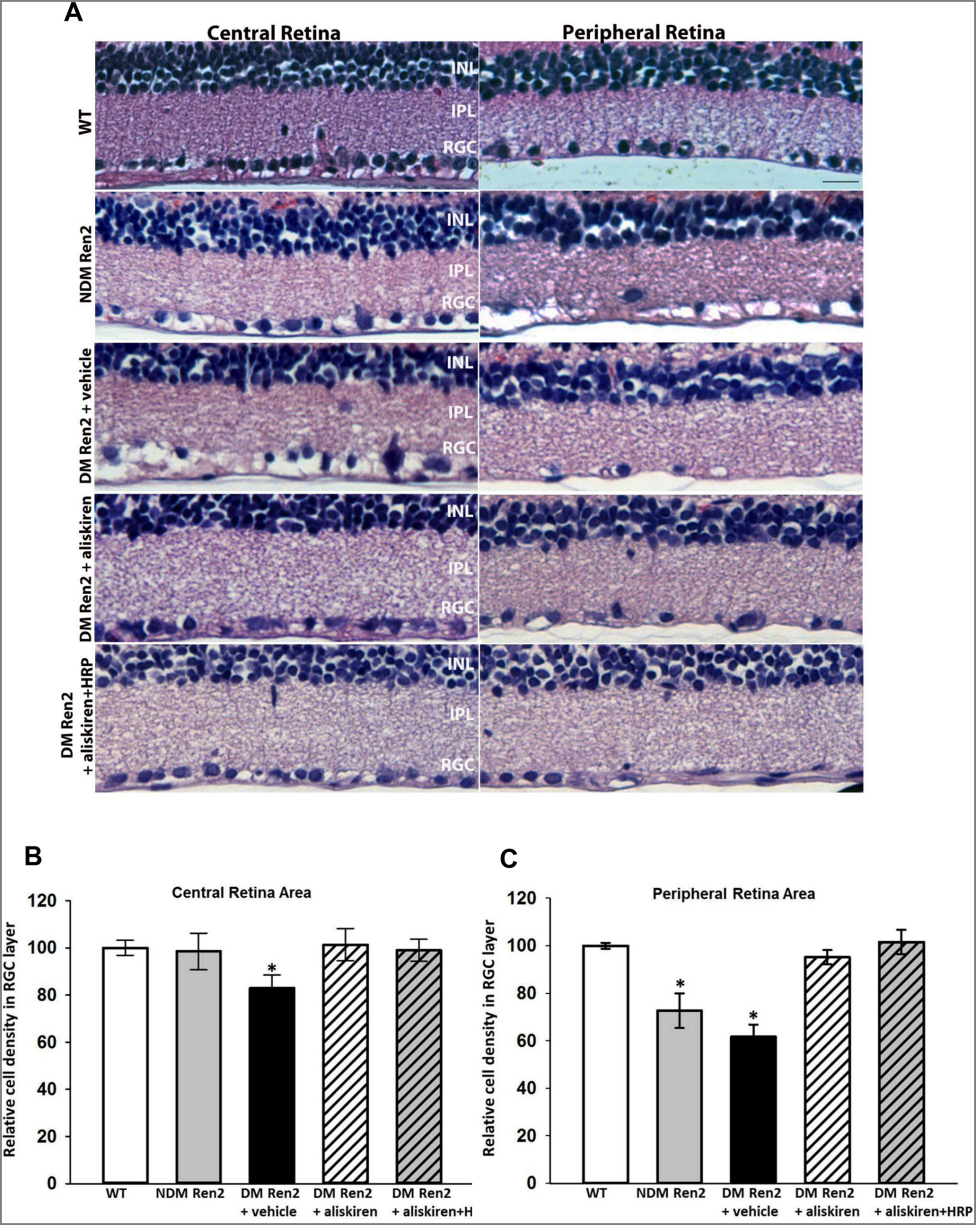
Representative retinal micrographs of wildtype (WT) Sprague–Dawley rats, non-diabetic (NDM) Ren2 rats, and diabetic (DM) Ren2 rats treated with vehicle, aliskiren or aliskiren+HRP (A). INL: inner nuclear layer; IPL: inner plexiform layer; RGC: retinal ganglion cell layer. Quantitative measurement of cell density in RGC layer in the central (B) and peripheral (C) retinas of wildtype (WT) Sprague–Dawley rats, non-diabetic (NDM) Ren2 rats, and diabetic (DM) Ren2 rats treated with vehicle, aliskiren or aliskiren+HRP. N = 4. *p<0.01 (vs. WT).

In the SD rat retina, very few apoptotic cells could be detected (data not shown). The Ren2 rat retina showed a significant increase of TUNEL positive apoptotic cells in both inner retina and photoreceptor layer (Figure 2a, b), and this increase was more prominent in the peripheral retina (Figure 2b) compared with the central retina (Figure 2a). This increase in apoptotic cells was dramatically further exacerbated in the diabetic Ren2 rat retina (Figure 2c, d). Treatment with either aliskiren alone or aliskiren+HRP significantly reduced apoptotic cell death to levels in the SD rat retina (Figure 2e, f, g). Morphologically the central retina of non-diabetic Ren2 rat is indistinguishable from wild type rat retina, however there is significant cell loss in the retinal ganglion cell (RGC) layer in peripheral retina (Figure 3). The diabetic Ren2 rat retina showed significantly increased cell loss in the RGC layer, which was more profound in the peripheral retina. The cell loss was normalized by either aliskiren or aliskiren+HRP treatment (Figure 3).

### Retinal capillaries

**Figure 4:**
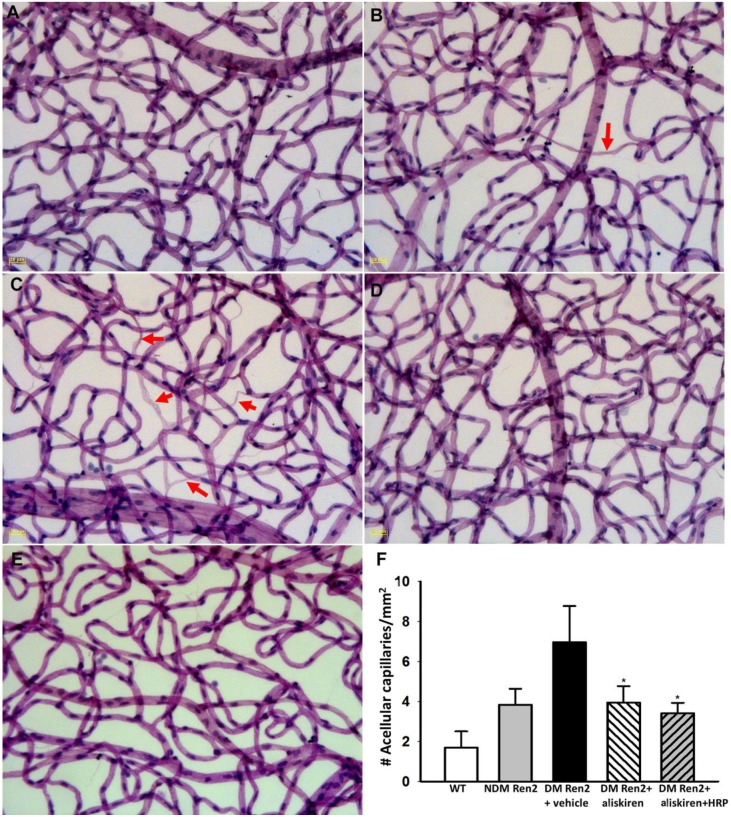
Representative capillary images of wildtype (WT) Sprague–Dawley rats (A), non-diabetic (NDM) Ren2 rats (B), and diabetic (DM) Ren2 rats treated with vehicle (C), aliskiren (D) or aliskiren+HRP (E). Diabetic Ren2 rat retinas showed further increased acellular capillaries (arrow) and both aliskiren and aliskiren+HRP reduced this. F: Quantitative measurements of acellular capillaries. *P<0.01 (vs. DM Ren2+ vehicle group).

The Ren2 rat retina showed an increased loss of capillaries compared to age-matched SD rat retinas (Figure 4A and B), and this loss was further exacerbated in diabetic Ren2 rat retinas (Figure 4C). Aliskiren reduced the acellular capillaries to the level seen in non-diabetic Ren2 rat retina (Figure 4D), but the number of acellular capillaries was still significantly higher compared to that of the SD retina. HRP treatment in combination with aliskiren did not add additional protection against capillary loss (Figure 4E). Figure 4F displays these results in a quantitative manner.

### Retinal inflammatory cytokines

**Figure 5:**
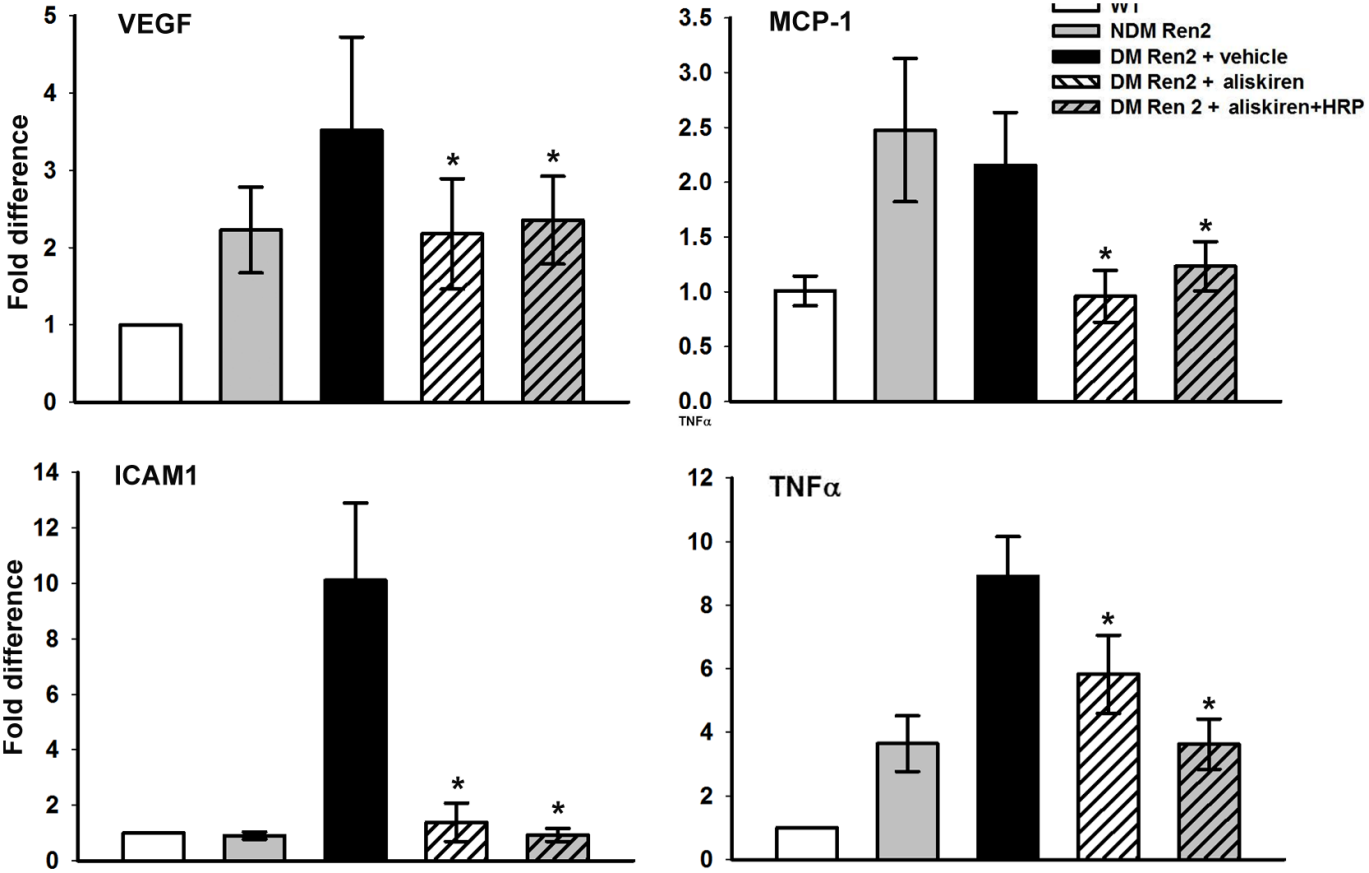
Real-time RT-PCR analysis of retinal expression of inflammatory cytokines: VEGF, MCP1, ICAM1 and TNFα. Values on y-axis represent fold change in mRNA levels compared to WT (normalized as 1). *P<0.01 (vs. DM Ren2+vehicle group). WT, wildtype, DM/NDM, diabetic/nondiabetic. N = 5.

Inflammatory cytokine expression in the retina was evaluated by real-time RT-PCT. VEGF expression was increased >2-fold in the Ren2 rat retina compared to age-matched SD rat retinas, and this increase was even larger in diabetic Ren2 rat retinas. Treatment with either aliskiren alone or combined with HRP reduced VEGF to non-diabetic Ren2 rat levels (Figure 5). MCP-1 expression was also significantly increased in Ren2 rat retinas with or without diabetes mellitus and aliskiren alone or in combination with HRP normalized this (Figure 5). A significant increase of ICAM1 was only seen in diabetic Ren2 rat retinas, and both aliskiren and aliskiren+HRP normalized this (Figure 5). TNFα expression was also highly increased in the Ren2 rat retina, and diabetes mellitus further upregulated this. Aliskiren significantly reduced TNFα to non-diabetic Ren2 rat levels. Aliskiren+HRP tended to reduce this even further, but the difference versus aliskiren was not statistically significant.

### Increased cytokine expression stimulated by recombinant prorenin in cultured human Müller glial cells and blockade by HRP and aliskiren

**Figure 6:**
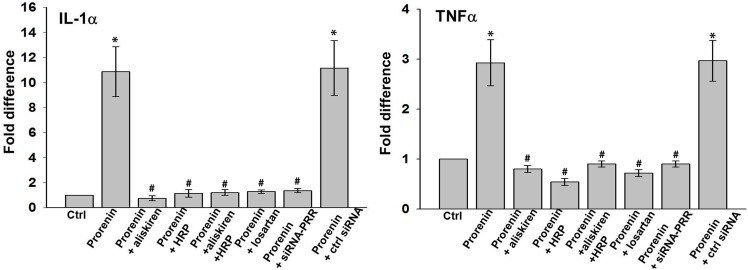
Real-time RT-PCR analysis of the expression of inflammatory cytokines IL-1α and TNF-α in cultured Müller cells. Values on y-axis represent fold change in mRNA levels compared to control (non-treated, normalized as 1). *P<0.01 (vs. control); # P<0.01 (vs. prorenin treated group). N = 4.

Müller cells, which span the entire neural retina, are the primary glial cell of the retina. They not only provide architectural support of the neural layers, but also have multiple functions such as providing metabolic support to energy-demanding retinal neurons, regulation of retinal synaptic activity by neurotransmitter recycling, and regulation of retinal water and ion homeostasis [48], [49]. Müller cells are intimately connected with retinal endothelial cells, pericytes, and astrocytes and play a crucial role to establish and maintain the blood–retinal barrier (BRB) [48], [49]. In addition, Müller cells modulate retinal immunity [50]. Moreover, Müller cells express several RAS components [7], [51], including (pro)renin [52] and its receptor (P)RR, so that Müller cell dysfunction might contribute to ocular dysregulation in diabetes [18], [21], [53], [54]. In fact, ocular prorenin upregulation is known to occur in patients with proliferative [10]–[12]. Prorenin was previously shown to increase proinflammatory cytokine expression in cultured neurons [55], To determine whether prorenin is also able to increase the expression of proinflammatory cytokines in Müller cells, cultured Müller cells were treated with prorenin, with and without HRP, aliskiren and/or the AT1R blocker losartan. The expression levels of IL-1α and TNF-α at 6 hours after the prorenin treatment were examined. Our results showed that the prorenin treatment significantly increased the expression of IL-1α (by ∼12-fold) TNF-α (by ∼3-fold) (Figure 6). Both aliskiren and HRP when given separately completely blocked these increases, and the results were identical when given in combination. Losartan treatment also completely blocked prorenin stimulated increased expression of IL-1α and TNF-α (Figure 6), suggesting that the increased expression of these cytokines is Ang II- dependent. (P)RR-siRNA completely blocked the prorenin stimulated increase of these cytokines, whereas the control scrambled siRNA had no effect (Figure 6), suggesting that the prorenin stimulated, Ang II-dependent increase in cytokine expression in Müller cells involves (P)RR binding.

## Discussion

The present study demonstrates that the Ren2 rat retina exhibited increased capillary loss and inflammatory cytokine expressions, and neuronal apoptotic cells. Diabetes mellitus further exacerbated these changes and enhanced gliosis. Renin inhibition counterbalanced these phenomena, whereas (P)RR blockade on top of renin inhibition did not offer additional advantages. The protective effect of aliskiren on retinal vasculopathy and inflammation in diabetic Ren2 rats is consistent with a previous report [56]. Diabetic retinopathy is not only associated with vascular dysfunction, but also with a loss of retinal neurons [57]. Our study further showed that aliskiren treatment normalized both photoreceptor and RGC loss in Ren2 rats to wildtype (SD rat) level.

Elevated plasma prorenin levels associate with microvascular complications in patients with diabetes mellitus. Prorenin is also highly elevated in ocular fluid in patients with diabetic retinopathy and its level correlates with the severity of DR in diabetic patients [10], [11]. Prorenin may contribute to diabetic complication and end-organ damage by binding to the (P)RR, resulting in increased generation of Ang II by the classic RAS pathway at the cell/tissue level [13]. Alternatively, such binding may initiate a cascade of signaling events that are associated with profibrotic and proliferative actions, independent of Ang II [13]. To what degree prorenin-(P)RR interaction is physiologically relevant, given the nanomolar prorenin levels (i.e., several orders above its normal plasma levels) that are required for such interaction, is still a matter of debate [58]. Nevertheless, since the eye is a prorenin-producing organ, with Müller cells releasing prorenin in the retina [52], if anywhere, such interaction might occur at ocular sites.

The importance of the (P)RR in the pathogenesis of end-stage organ damage in diabetes and hypertension has been demonstrated by in vitro and in vivo animal studies using HRP, the only (P)RR blocker that is currently available [15]. The possible pathological role of the PRR in the retina has been established by several reports in which HRP was shown to prevent pathological angiogenesis [18], [21], and ocular inflammation induced by endotoxin [20] and diabetes [19]. It was thus expected that blockade of both the Ang II-dependent pathway (by aliskiren) and the (P)RR-mediated, Ang II-independent pathway (by HRP) would provide better protection against diabetes-induced retinal pathophysiology. However, the present study showed that HPR did not provide additional beneficial effects on top of aliskiren. In part, this is due to the fact that, with regard to at least some parameters, aliskiren already provided maximal benefit. This would suggest that prorenin-dependent effects, if present, might be largely due to activation of the Ang II-AT_1_ receptor axis. Obviously, aliskiren will block angiotensin generation by receptor-bound prorenin (or renin) [31], [32]. In addition, renin inhibition may lower (P)RR expression [33], thus potentially diminishing the capacity of HRP to exert an effect at all. It is unlikely that aliskiren blocked renin/prorenin-(P)RR interaction, since renin/prorenin binding to the (P)RR was unaltered in the presence of aliskiren, while aliskiren also did not affect (pro)renin-induced signaling [58], [59].

We previously reported that aliskiren normalized vascular dysfunction in diabetic hypertensive rats, while HRP unexpectedly counteracted these beneficial vascular effects in a blood pressure- and angiotensin-independent manner [35], [36]. This may be explained on the basis of partial agonism of HRP towards the (P)RR [21], [60]. Intriguingly, although HRP did not exert beneficial effects on top of aliskiren in the diabetic Ren2 rat retina, it also did not counteract the protective ocular effects of aliskiren. Thus, possibly the retinal levels of HRP (when administered systemically) were not sufficiently high to generate similar counteracting effects. Yet, when infused at similar doses alone, HRP did exert beneficial effects in the diabetic eye [21]. It cannot be excluded that such effects are (P)RR-independent, but if so, they can apparently no longer be seen on top of RAS blockade.

Hypertension plays an important role in retinal pathophysiology and is one of the major risk factors that exacerbates the initiation and progression of DR [1]. As shown in our previous report [36], diabetic Ren2 rats displayed severely elevated blood pressure; aliskiren normalized blood pressure, suppressed renin activity, and reversed vascular dysfunction, whereas HRP did not affect the blood pressure-lowering effects of aliskiren [15], [35], [61]. The beneficial effects of aliskiren in the diabetic Ren2 rat retina may be due to its blood pressure-lowering effect and associated improvement of renal and other systemic cardiovascular function. However, in a previous study in the same model, ACE inhibition resulted in better blood pressure control, while aliskiren provided greater retinal protection [56]. This strongly suggests that the beneficial effects of renin inhibition extend beyond the blood pressure-lowering effects of aliskiren. Clearly, this needs to be tested in normotensive animal models of diabetic retinopathy in future studies.

In cultured Müller cells, prorenin significantly increased the expression levels of IL-1α and TNF-α, consistent with previous observations [55]. Both aliskiren and HRP completely blocked these effects as did treatment with (P)RR siRNA and the AT1R blocker losartan. This suggests that these prorenin-induced effects are entirely (P)RR- and Ang II-dependent, i.e., involve Ang II generation by (P)RR-bound prorenin, identical to the prorenin-dependent Ang II generation described earlier in vascular smooth muscle cells [58]. Possibly, such generation also contributes to the elevated ocular Ang II levels under many pathological conditions [10]–[12], [51], [62]–[64].

Recent evidence clearly indicates the (P)RR as multi-functional receptor, affecting a wide range of signaling pathways, many of which are not necessarily related to prorenin and Ang II/AT1R signaling. In fact, a truncated form of (P)RR at the C-terminal region was previously described as a vacuolar H^+^ -ATPase (V-ATPase) associated protein (Atp6ap2), and the (P)RR may thus also affect V-ATPase activity [65]. Such activity determines the intracellular pH homeostasis that is critical in diverse physiological cellular functions including endocytosis, processing of proteins and signaling molecules, membrane sorting and trafficking, activation of lysosomal and autophagosomal enzymes, and patterning during development [66], [67]. Consequently, it is conceivable that the (P)RR contributes not only to tissue RAS activation but also to multiple molecular and cellular processes via its V-ATPase connection. Further studies are required to further elucidate whether prorenin affects these functions.

In conclusion, the renin inhibitor aliskiren reduced both neuronal and vascular pathologies, including retinal gliosis, apoptotic cell death of retinal neurons, acellular capillaries and expression of inflammatory cytokines in diabetic mRen2 rats. The putative (P)RR blocker HRP did not provide add-on beneficial effects when combined with aliskiren, nor counteracted the effects of aliskiren in the retina of diabetic mRen2 rats. The latter contrasts with earlier observations in the kidney of diabetic mRen2 rats, where HRP counteracted the beneficial effects of aliskiren [47]. The former agrees with our in-vitro studies in Müller cells, showing prorenin-induced effects that are entirely Ang II-dependent. Taken together, these results suggest that aliskiren is a promising treatment option for patients with diabetic retinopathy, and that its combination with HRP in this condition is not advisable.
